# Land Tenure and Cotton Farmers’ Land Improvement: Evidence from State-Owned Farms in Xinjiang, China

**DOI:** 10.3390/ijerph19010117

**Published:** 2021-12-23

**Authors:** Bo Li, Ruimei Wang, Quan Lu

**Affiliations:** 1 College of Economics and Management, Tarim University, Alar 843300, China; luquan0122@163.com; 2 College of Economics and Management, China Agricultural University, Beijing 100083, China; 3 College of Economics and Management, Huazhong Agricultural University, Hubei 430077, China

**Keywords:** land tenure, land improvement, dynamic model, IV ordered probit, conditional mixed process (CMP), control function (CF), cotton farmers, intergenerational transfer of land tenure, non-property factors

## Abstract

The land system of state-owned farms in China is different from that in rural areas. Whether the land tenure of state-owned farms can play a role in protecting cultivated land is an important issue for the high-quality development of state-owned agriculture in China. This article develops a dynamic model to examine how land tenure influences farmers’ decisions on land improvement. It then analyzes this relationship based on cotton farmers’ household-level data from state-owned farms of Xinjiang in China. We applied methods that take into account the possible endogeneity of the land tenure. The results reveal that the stability of land tenure in the past will not affect the current behavior of farmers for they have a relatively stable expectation of current land tenure and a high degree of trust in the government and its policies. The intergenerational transfer of land tenure is not the key factor that affects farmers’ land conservation, and the relatively long-term duration of land tenure (possibly five years or more) during their careers is more important. Our findings also reveal that non-property factors, such as government intervention (e.g., technology promotion) that alleviates the limited rationality of farmers, cannot be ignored because they played a crucial role in past land improvement when land tenure was less stable.

## 1. Introduction 

In 2017, China proposed that its economy should shift from high-speed growth to high-quality development. To promote the high-quality development of agriculture, special attention should be paid to the protection of cultivated land resources and the promotion of high-quality development of cultivated land. Improving land productivity and crop quality by ensuring the soil health of cultivated land should ultimately contribute to better quality and efficiency in agriculture. As early as 1987, the Chinese government incorporated the protection of cultivated land quality into law. Since 1988, about 16 provinces (cities) in China have promulgated special regulations, measures or regulations on the protection of cultivated land quality. However, according to the Ministry of Land and Resources of China’s 2016 arable land quality survey (China, Ministry of Land and Resources. Announcement on the main data results of the national cultivated land quality grade update evaluation in 2016, http://www.gov.cn/xinwen/2017−12/28/content_5251076.htm (in Chinese))(accessed on 28 December 2017), the quality of arable land declined compared with 2008, and the proportion of medium and low arable land increased [1]. This shows that the quality level of cultivated land in China has declined in a relatively long historical period, and there is still a gap between the status quo of cultivated land conservation and the expectation of policy.

The users of cultivated land are the core subjects of cultivated land conservation, and rational small-scale farmers are the logical starting point. Whether farmers adopt the practice of cultivated land conservation depends on the measurement of cost and benefit [2]. Farmers will not take action if the cost of maintaining their land outweighs the potential benefits. Investment in cultivated land conservation is not a short-term investment, in which farmers invest capital, time, or labor costs in the current year and obtain full returns in subsequent years. If the land tenure is not stable and lacks external incentives and economic compensation, the peasant household will either reduce the related investment or not even invest. Therefore, to encourage farmers to maintain cultivated land, in the absence of external incentives and compensation, we should first ensure that they have stable property rights and a longer planning period.

The role of land tenure on investment in conservation agricultural measures has been widely documented in the relevant literature. The mechanism of stable land tenure influencing agricultural investment through income effect (stabilizing expected income), transfer effect (promoting efficient circulation), and mortgage effect (increasing the possibility and value of the mortgage) has been agreed upon [3,4]. Many studies have shown that stable land tenure tends to have positive effects on long-term investments in most cases [5,6,7,8]. There is, however, a considerable amount of conflicting evidence. Knowler et al. (2007) concluded 31 papers on farmers adopting conservation agriculture and found little analysis to support the hypothesis that owned land is better maintained than leased [9], and Prokopy et al. [10] reached a similar conclusion. 

Moreover, the institutional background as well as land markets differ substantially from different countries, so the focus on land property rights is different [11,12,13,14,15]. Developed countries are concerned about the instability caused by land lease [10,16,17,18,19,20,21]. Developing countries, such as Africa, are concerned about the instability caused by imperfect laws or involuntary adjustment of land [3,5,7,22,23,24,25,26,27]. Therefore, it is unlikely to converge toward a particular universal explanation due to different contextual variables. The development of different national supporting systems determines whether land property rights can play a role in farmland protection.

Under the separation of land property rights in China, the previous research on land tenure mainly focused on rural areas and less on the land tenure of state-owned farms. Previous studies mainly focused on rural areas, examining the impact of involuntary land adjustment, policies to stabilize land property rights, and land transfer on land investment [4,6,28,29,30,31]. However, in addition to rural collective-owned agricultural land, there are about 160 million *mu* of state-owned agricultural land on state-owned farms in China (China had 1843 state-owned farms in 2019, of which Xinjiang, Jiangxi, Heilongjiang, Fujian, Inner Mongolia, and Liaoning had more than 100 farms, while Xinjiang had the highest number of state-owned farms and workers). Compared with the rural areas of China, the state-owned farms have obvious advantages in scale and organization, which is also the direction of agricultural reform in rural areas. The land policy of state-owned farms in different regions is different due to the lack of special laws and regulations and the low level and small quantity of local regulation of land relations. Under the background of the “three rights division,” rights are divided into ownership rights, contract rights, and management rights. Usually, ownership belongs to the village collective, while the contract rights belong to farmers belonging to this village. Farmers with contracting rights can transfer the land management rights to any person or organization. State-owned farms also actively promote the division of rights and interests. Unlike property rights in rural areas, land on state-owned farms is owned by the state, which the farms have the right to use, and the farm workers have the right to contract for the management of land, and the right to operate can gradually be owned by any individual or organization (this may vary from farm to farm).The characteristics of land tenure owned by farm workers are similar to those of rural farmers. However, there are still some differences between the state-owned farm workers and rural farmers in terms of the land contract term, land lease term, and scope.

This study will examine the effect of land tenure on cotton farmers’ land improvement behaviors from the state-owned farms in Xinjiang, which is also called Xinjiang Production and Construction Corps (XPCC). The XPCC is a large, state-owned farm with different levels of farms under its jurisdiction. Before 1978, the land of the XPCC was owned by the state, and each XPCC farm had the right to use it. After 1978, based on the household responsibility system (HRS), the XPCC gradually established a two-tier management system of large farms and small farms; that is, the management rights are jointly owned by the group farm and its employees who mainly work on the land. However, farmers did not have sufficient autonomy and needed to plant under unified arrangements. In 2006, the XPCC implemented the long-term land fixation policy, stipulating that the maximum period of contracted land is 30 years, and wasteland can be 50 years, but the contract needs to be signed in installments (usually five years). It further clarified and expanded the autonomy in production and management of contracted farmers. In 2017, the XPCC carried out land readjustment and land titling, with land distributed evenly and contracts limited to the retirement age. The retirement age is usually 60 for men and 50 for women. By abolishing the “five unifications,” which are restrictions mainly aimed at cotton farmers and mainly refer to unifying planting plans, unifying agricultural materials procurement, unifying product purchases, unifying agricultural machinery operation levels and charging standards, and unifying technical guidance, farmers’ self-management is greatly strengthened, and they become the real rational economic man. 

Cultivated land in the XPCC can be divided into "quota land," "business land," and "own-use land". "Quota land," also known as "identity land," which is enjoyed according to the employee status of the XPCC farm, is mainly used to meet the basic needs of farmers and pay social insurance. Before the reform in 2017, it was stipulated that the contract period could not exceed 30 years, and the contract was signed with employees in installments (usually five years). After the reform, the contract period is only limited to the retirement age. "Business land" is market-oriented land and is available to anyone willing to expand their acreage, but leases are usually for a period of one to three years. This part of the land cannot be fixed for a long time, nor can it be transferred. "Own-use land " is used to grow vegetables for self-sufficiency or to develop the courtyard economy in front of and behind their house. Under the 2017 land reform, corps farms are required to give each employee no less than 40 mu of “quota land,” depending on local land resources. If a farm household has two employees, the farm household has at least 80 mu of land.

The land system of state-owned farms in Xinjiang is different from that of rural areas in China, it is necessary to explore the role of land tenure in the protection of cultivated land. First, in addition to state-owned land, rural land belongs to the village collective ownership, the village farmers enjoy usufructuary rights. However, the land of the XPCC belongs to the state, and the regiment farm exercises the management rights of the land under its jurisdiction, and the farmers of the farm enjoy the right of use. Secondly, before the land system reform in 2017, farmers in the XPCC had limited autonomy in production and operation. The farm implemented the "five unifications" and only transferred part of the land use right. The contract is signed every one to five years, and the benefits and risks are shared with the farm. After land reform in 2017, its operational autonomy is no different from that of farmers in other rural areas of China. Thirdly, unlike other rural areas in China, the duration of farming contracts for XPCC farmers is limited to the retirement age. If the contract is terminated for various reasons, the land may first be contracted to the eligible spouse or children. Finally, what is more special is that the XPCC’s transfer of land contractual management rights are limited to a certain extent. The XPCC only allows the transfer of identity land between the contracted employees of the regiment farm; that is to say, it cannot be transferred to individuals or organizations outside the regiment farm. Overall, since the land reform in 2017, the rights of peasants in the XPCC have gradually converged with those of peasants in other rural areas, but there are some differences in the nature of land property rights, term of use, and land transfer. As far as land tenure is concerned, the land tenure of the XPCC is less stable than those of rural areas because of the relatively frequent land readjustment and the shorter term of land tenure. The XPCC limits the duration of land contracts to retirement age. If a farmer has two years to retire, he has only two years to farm. So, in this context, what role does land tenure play in promoting farmers to conserve cultivated land?

The contributions of this paper are as follows: (1) This paper contributes to the current literature on land tenure and land improvement of cotton farmers by developing a dynamic optimization model. We selected land tenure variables based on the regional land system and discussed land improvement measures consistent with local cotton agronomy and extension trends. Although the land tenure system and land improvement practices in the study area have certain particularities, it is still helpful to provide new evidence and general conclusions. (2) Through the discussion of the relationship between land tenure and land improvement, it is helpful to clarify the role of land tenure in cultivated land conservation, and further optimize the state-owned farm land tenure systems and cultivated land conservation supporting systems. (3) Many scholars in China put forward the idea of endowing farmers with “permanent right of use” and “inheritance right” from the perspective of encouraging farmers to use and maintain their land [32,33]. This paper attempts to answer the question of whether or not the relatively long-term tenure and the children continue to contract is conducive to the conservation of cultivated land, so as to provide a theoretical basis for the perfection of China’s land system. 

## 2. Methodology

### 2.1. Theoretical Analysis

In line with previous studies, such as McConnell [2], Gebremedhin and Swinton [7], and Ma et al. [34], we constructed a model of land improvement decisions from the perspective of land tenure. Assuming that farmers produce only one crop, the profit function of a single crop is:

(1) $$\pi =pgf\left(s,x\right)-c_{1}x-c_{2}z,\;$$

where p represents crop prices, g represents neutral technical progress, x represents productive inputs, and z represents land improvement investments. $c_{1}$ and $c_{2}$ are price indices for productive inputs and land improvement investments, respectively. Soil quality, s, means that land input and land element quality are equally important to farmers’ output, which is subjected to the transition equation:

(2) $$\dot{s}=h\left(x,z\right),$$

where *h* represents the change in soil quality. Soil quality here refers primarily to soil fertility, that is, the ability of soil to provide plant nutrients and produce biological substances. 

Productive inputs reduce the soil quality (*∂h/∂*x ≤ 0), and land improvement investments increase soil quality (∂*h*/∂z > 0). Land improvement measures are not necessary inputs for crop growth; they may be additional capital or labor inputs, such as the application of organic fertilizers, soil amendment, microbial fertilizers, or deep pine. Meanwhile, they may also be a change in production mode, such as changing from empirical fertilization to formula fertilization or from applying inorganic fertilizers to chemical fertilizers with higher organic matter. 

Furthermore, land improvement inputs may either replace or complement other input elements. For example, if farmers switch from empirical to formula fertilization, the use of fertilizer will be reduced in the case of excessive inputs. With the increase of the input of organic fertilizer and the potential of cultivated land, farmers can increase the yield of crops by putting more fertilizer in the case of insufficient fertilizer, but in the case of excess fertilizer, farmers can reduce the fertilizer to achieve the target yield.

In any case, as the investment in land improvement increases, soil quality will be improved (∂h/∂z > 0). Yield increases as soil fertility increases, but ultimately, additional investment in land improvement does not increase yield. When the nutrients in the soil are saturated, it is impossible to increase yields by continuing to invest. Therefore, we expect that $f_{s}>0$ and $\;f_{ss}<0$.

Farmers’ decisions to improve the soil depends on the impact of soil quality on their profits. Assume that the farmer works his land to maximize the net present value of crop income and farmland value throughout the planning period. And suppose that farmers have perfect foresight about the knowledge of various technical and economic relationships. Let the terminal value be *R(s)*, where *R* stands for the resale value of the farm. Making *R* a function of *s* implies that the value of farmland depends on soil quality. The present value of profits for *T* years is

(3) $$J=\int_{0}^{T}e^{-\gamma t}\left[pgf\left(s,x\right)E\left(T_{t}\right)-c_{1}x-c_{2}z\right]dt+R\left(s\right)e^{-\gamma t}$$

(4) $$s.t.\;\;\dot{s}=h\left(x,z\right)\;s\left(0\right)=s_{0}\;$$

where γ is the discount rate, and $E\left(T_{t}\right)$ is the expected value that land tenure will be retained in period *t*. This expectation is assumed to be binary and non-switching, such that the farmer either expects (*E*(*T_t_*) = 1) or does not expect (*E*(*T_t_*) *=* 0), and it cannot be regained in a later period. 

The undiscounted Hamiltonian associated with (3) and (4) is:

(5) $$H=\left[pgf\left(s,x\right)E\left(T_{t}\right)-c_{1}x-c_{2}z\right]+\lambda h\left(x,z\right)$$

λ is the undiscounted Hamiltonian multiplier, which represents the marginal value of the state variable *s* at time *t*, also known as the shadow price of soil quality. Equation  λ(T)=∂R(s)/∂s shows that it is not economical for a farmer to exhaust the value of his farm at the end of his career when the fertility of arable land can be well priced.

According to the maximum principle, the optimal condition equation satisfies:

(6) $$\partial H\left(x,z,s,\lambda \right)/\partial z=\left[pgf_{s}\left(s,x\right)h_{z}E\left(T_{t}\right)-c_{2}\right]+\lambda h_{z}\left(x,z\right)=0,$$

(7) $$pgf_{s}\left(s,x\right)h_{z}E\left(T_{t}\right)+\lambda h_{z}\left(x,z\right)=c_{2}.$$

Equation (7) specifies that optimal land improvement investment occurs where the marginal cost of land improvement equals the crop revenue and land improvement value. This optimality condition highlights the importance of the subjective expectation of enjoying land tenure in period *t* ($E\left(T_{t}\right)$). The expectation of land tenure dictates the length of the planning horizon, thereby largely determining whether farmers will improve the land or not. Compared with those who have unstable tenure, farmers with stable land tenure will take land improvement measures. In addition to crop yields, improving the marginal value of land quality can also increase farmers’ investment in land improvement, which comes from reasonable government compensation or good market pricing.

### 2.2. Data and Sample Description

#### 2.2.1. Data

The primary data of this study were collected in May 2020 in the First and Seventh Divisions of the XPCC. The First Division and Seventh Division are two divisions with larger cotton planting areas in Southern Xinjiang (south of the Tianshan Mountains) and Northern Xinjiang (north of the Tianshan Mountains). Their acreage ranks second and third among the corps’ 14 divisions, respectively. The stratified sampling data of 1287 cotton farmers were collected by random sampling method. Since each household only gets the data of the largest plot, and the land transfer is not considered in this study, a total of 1031 contracted plots were obtained.

China’s cotton production areas are mainly concentrated in Xinjiang (including the XPCC), Hebei, Shandong, Hubei, and Anhui. The five major production areas occupy 93% of the area and contribute 95% of the production. With the adjustment of planting structure in the Yangtze River basin and the Yellow River basin, the cotton sown area (including the XPCC) in Xinjiang was gradually expanded to 74% in 2018, accounting for 84% of the national cotton output. It has become the largest cotton-producing area in China. Among them, the cotton planting area and yield of the XPCC accounted for one third of the total area and yield of Xinjiang (including the XPCC). (Source: *Statistical Yearbook of Xinjiang Production and Construction Corps in 2019*.)

According to the report on cultivated land quality grades in Xinjiang in 2020, the average grade of cultivated land in Xinjiang is lower than the national average. Saline-alkali soil, barren soil, desertification, and poor basic land capacity are the main obstacle factors. In addition, Xinjiang is the largest area covered by plastic film, but also the most serious areas of soil “white pollution”. Under the situation of poorly cultivated land foundation and serious cultivated land pollution in Xinjiang, how to reduce the cultivated land pollution and improve the cultivated land production potential are very important for the local agricultural economic development and the protection of cultivated land resources.

#### 2.2.2. Sample Description

In terms of land readjustment (Table 1), 38.41% of the farmers have not experienced land readjustment, 29.29% of the farmers have experienced land readjustment once, and 32.3% of the farmers have experienced land readjustment twice or more. As can be seen, land tenure instability is common. In the last land readjustment (2018), the proportion of farmers whose area changed was the highest at 69.19%, while 12.86% of farm plots changed, and only 28.07% remained unchanged. 

According to the latest land contract regulations, when a farmer terminates the contract for various reasons, the land can be optimally contracted to his or her eligible spouse or children. In the sample, 34.53% of farmers will hand over the land to their children to continue farming when the contract expires, and 60.33% of farmers will return their land to the farm. In addition, the XPCC has the advantage of scale and a high commercialization rate in cotton planting. The farmers are generally young, and 58.49% of them are expected to farm for more than 10 years.

The land improvement measures of cotton farmers in Xinjiang mainly include the application of organic fertilizers (such as fermented chicken manure and sheep manure), soil amendments, compound fertilizers with high organic matter, and microbial fertilizers, as well as soil testing formula fertilization, deep pine, and crop rotation. All the above actions were chosen by the farmers themselves.

Table 2 shows that 45.39% of farmers improved their land before the last land readjustment (before 2017), and 50.05% improved their land after the last land readjustment (after 2017). Overall, 51.70% of farmers improved their land in the past. Furthermore, 96.37% of farmers who improved their land before 2017 still improved their land afterward. A total of 82.24% of farmers who had not improved their land before 2017 remained on the sidelines after the last land readjustment. Only 11.55% of farmers who had not previously improved their land improved their land afterward. There is productive inertia in land improvement, and farmers’ behavior has a certain sustainability. Once a farmer has improved his land in the past, he is more likely to improve his land in the following years. This may be due either to the fact that farmers are aware of or have benefited from the improvements or to the fact that the production uncertainty that may result from a reduction in such inputs, which makes farmers willing to continue to invest in order to avoid risks.

As for the land improvement measures chosen by farmers (Table 3), the proportion of organic fertilizers was the highest (60.79%), followed by compound fertilizers with higher organic matter (37.52%), formula fertilization (36.02%), and microbial fertilizers (34.52%). The application of soil amendments (19.51%) and other improvement measures, such as deep pine or rotation (15.01%), are relatively low. Furthermore, 48.22% of farmers adopted one of the land improvement measures, and 21.76%, 16.32%, and 13.69% of the farmers adopted two, three, and four or more land improvement measures, respectively. In terms of regional differences, the proportion of organic fertilizer used in the First Division was 12.58% higher than that in the Seventh Division, while the proportion of the Seventh Division using microbial bacterial fertilizer and compound fertilizers with the higher organic matter was 14.05% and 21.72% higher than that of the First Division. The proportion of other measures adopted is similar.

### 2.3. Empirical Specification

#### 2.3.1. Basic Model

We mainly examined the impact of land tenure security on farmers’ land improvement. We let Y denote the farmer’s behavior of land improvement, as it is a three-category discrete variable: 2 = land has improved after the latest land adjustment in 2018; 1 = under consideration; 0 = land has not improved after the latest land adjustment in 2018. These observable values Y came from some unobservable latent variables $Y^{*}$:

(8) $$Y^{*}=\alpha T+\beta X+\varepsilon$$

(9) $$Y=\{\begin{array}{cc} 2, & \;\;if\;\;\mu_{1}<{\;Y}^{*}\leq \mu_{2\;}\\ 1, & –if\;\mu_{0\;}<{\;Y}^{*}\leq \mu_{1\;}\\ 0, & if\;Y^{*}\leq \mu_{0\;} \end{array}$$

where T is defined as land tenure. We chose three discrete variables to characterize land tenure security: *the number of land adjustments* ($T_{1}$), *remaining term* ($T_{2}$), and *child farming* ($T_{3}$). “The number of land adjustments that the peasant household has experienced since contracting land” represents the variable *number of land adjustments* ($T_{1}$). The more land adjustments, the more unstable the land tenure of farmers in the past, and the lower the possibility of farmers improving the land. “Whether the remaining farming years are more than 5 years” represents the variable *remaining term* ($T_{2}$), and if the remaining years of farmers exceed 5 years, it means that farmers have relatively long-term property expectations, and their land tenure is relatively stable. “Whether the children continue to farm after the contract expires” represents the variable *child farming* ($T_{3}$), which represents the farmers’ expectation of intergenerational land tenure transfer. Land tenure is expected to be more stable if children continue farming beyond the end of their contracts. According to the theoretical analysis,$\;T_{2}$ and $T_{3}$ are predicted to be positively correlated with farmers’ land improvement, while $T_{1}$ has a negative correlation. Then, X is the vector of distinct exogenous variables, and ε is assumed to follow the standard normal distribution. Equation (8) is estimated by ordered probit regression.

However, many scholars have considered the endogenous problem of land tenure and farmland investment in related studies, that is, the more stable the farmers expect to farm, the more likely they are to improve their land. Conversely, if farmers are considering land improvement, they may have relatively stable land tenure [4,23,26]. Technically, the endogeneity arises due to the presence of the correlation between $T^{*}$ and ε, which may bias the estimate of $\hat{\alpha }$. Since $T_{1}$ and $T_{2}$ are predeterminate variables, the endogenesis of this paper mainly exists between $T_{3}$ and Y. 

In order to solve the endogeneity problem, we chose the mean value of the land tenure left to the children of the farm households as the instrumental variable of $T_{3}$. The transfer of land tenure from other farmers to their children does not directly affect whether the farmer improves the land, but it does affect the possibility of the farmer transferring land tenure to the farmer’s children. Since farming is not a popular occupation, farmers may prefer their children to work in relatively decent jobs, but if a larger number of farmers in the farms prefer to transfer their land tenure to their children, the impact will be mitigated.

#### 2.3.2. Estimation Strategy

To test potential reverse causality, a Two-Stage Least Squares method (2SLS) will produce consistent estimates of the parameters. However, when the dependent variable is discrete, the 2SLS approach will not be able to address the endogeneity problem. To obtain a consistent $\hat{\lambda }$, we considered the IV ordered probit model and control function to deal with the endogeneity issue.

First, we used the two-step method of Heckman [35] to estimate the parameters. In the first stage, we used a binary probit model to regress the endogenous explanatory variable *T* on the instrumental variable and the exogenous explanatory variable to obtain the fitting value of the latent variable $T^{*}$, namely:

(10) $$T^{*}=\delta Z+\theta X+u$$

(11) $$T=\{\begin{array}{c} 0,\;{\;T}^{*}<0\\ \;1,\;{\;T}^{*}\geq 0\; \end{array}$$

(12) $$\hat{T^{*}}=\hat{\delta }Z+\hat{\theta }X$$

where T is a dummy variable, ^ is the fit value of the variable or the estimate value of the parameter, X is a vector of control variables, and Z is the instrumental variable. In the second stage, the latent fitting values $\hat{T^{*}}$, exogenous explanatory variable X and residuals are regressed by $Y^{*}$ in order probit regression.

(13) $$Y^{*}=\alpha^{*}\hat{T^{*}}+\beta X+\varepsilon$$

A consistent estimate of $\alpha^{*}$ can be obtained from the two-stage regression. 

Further, we tested the robustness of the IV ordered probit approach using the Conditional Mixed Process (CMP) and control function (CF) suggested by Roodman [36] and Wooldridge [37], respectively. The CMP is a SUR estimator; it can be used to estimate the multistage regression model by constructing the recursive equations based on the maximum likelihood. In particular, there are obvious advantages in the estimation of mixed models with class variables or censored data variables as endogenous variables. This procedure enabled us to assess whether the correlation between the error terms in Equations (8) and (10) are equal to zero. The failure to reject this hypothesis implies the exogeneity of tenure, and thus a standard ordered probit regression would suffice.

The CF approach is inherently an instrumental variables method, which can be applied to various linear and nonlinear models and handles fairly complicated models that are nonlinear in endogenous explanatory variables. By adding appropriate control functions, which are usually estimated in a first stage, the endogenous explanatory variables (EEVs) become appropriate exogenous in a second-stage estimating equation. However, CF methods for the estimation of the nonlinear model with discrete EEVs are more controversial because they rely on nonstandard assumptions. Rather than using the predicted values of the endogenous variable obtained through the first-stage regression, the CF approach introduces the residuals obtained from the first-stage equation, and the endogenous variable itself, into the second-stage equation. Specifically, we can rewrite Equation (8) and Equation (10) as:

(14) $$Y^{*}=\alpha T+\beta X+\lambda \hat{u}+\varepsilon$$

(15)  T=δZ+θX+u

For Equation (15), a linear probability model (LPM) is computed and residual extracted. The residual ($\hat{u}$) obtained from this equation, together with the EEVs, are then introduced into the Equation (14), which is an ordered probit model. Here, the significance of λ is the criterion used to test for the exogeneity of land tenure; if λ does not significantly differ from zero, there is no simultaneity bias. Technically, this CF approach is also called 2SCML (Two-Stage Conditional Maximum Likelihood), which was developed by Rivers and Vuong [23,26,37,38].

#### 2.3.3. Control Variables Selection

We selected the factors that affect farmers’ land improvement, including basic family characteristics and cotton planting characteristics, as well as economic, technical, and institutional factors directly related to land improvement. The meaning, description, and prediction of variables are shown in Table 4. The prediction of the influence direction of the variable can refer to the studies of Knowler and Bradshaw [9], Adimassu et al. [8], Prokopy et al. [10], and Foguesatto et al. [39].

*(1) Basic family characteristics.* Basic family characteristic variables include age, education, grassroot cadres, and risk attitude, which are usually used as cultural proxy variables to infer the social and cultural reasons behind the behavior of farmers [40,41]. When the household head ages, this means that his career in farming is about to end, and his motivation for improvement will decrease. The higher the family’s highest educational background, the stronger the family’s knowledge and technology acceptance, and the more helpful it is for farmers to take rational actions to improve the land. If there are cadres in the family, the stronger their ability to obtain technical or policy information for land improvement, the higher the possibility of land improvement. The riskier farmers are, the more likely they are to adopt long-term investment measures. 

*(2) Cotton planting characteristics.* Variables that are highly associated with cotton production include experience, the area planted, specialization, and location variable. Generally speaking, the longer the cotton is planted, the more farmers have the relevant knowledge and technical experience, and the more likely they are to improve the land. However, rich planting experience also means that farmers’ careers in farming are coming to an end, and the possibility of improving the land is less. Therefore, the impact of the planting experience is uncertain. Compared with small-scale farmers whose main goal is to survive, large-scale farmers have a stronger motivation to improve the land to obtain higher returns. The higher the degree of specialization in cotton production, the stronger their business awareness, and the greater the possibility of land improvement.

*(3) Economic factors.* Economic factors are the basis of farmers’ land improvement [2,42]. These include profitability, income, and financial constraints. Farmers can perceive or observe that higher relative returns will increase farmers’ land improvement investment [43,44], so it is predicted to be positively correlated with farmer behavior. Family income affects the participation ability and willingness of farmers. On the one hand, it is difficult for subsistence farmers to change their farming methods due to their limited ability. On the other hand, economic pressure makes farmers more willing to tolerate unethical behavior [45]. The better the family economic foundation, the smaller the capital constraints of farmers, which may be positively related to farmers’ behavior. Furthermore, if farmers have no financial constraints, it means the liquidity constraints faced by farmers are relatively small, and their investment behavior will not be restricted, so the possibility of land improvement is greater.

*(4) Technical factors.* To encourage farmers to adopt related conservation technologies, it is necessary to ensure that farmers can learn and develop related technologies or obtain related technical services [46]. In the process of land improvement, there are complex technical issues about how much organic matter is applied, and how and when to apply it. Even if the land improvement is considered valuable, if farmers have technical constraints, the possibility of improvement is relatively small. Related variables include government technical assistance, enterprises services, technology constraints, and technical experience. If farmers have received technical guidance from the government or company, there are fewer technical constraints, and the possibility of investing in land improvement is greater. If farmers have experience in using technologies to improve their land, they are more likely to continue using them.

*(5) Institution factors.* The institution has the function of incentive and constraint, which can change the transaction cost of farmers participating in environmental governance. Effective institutional arrangements are the guarantee for farmers’ participation [47]. The institutional factors in this article mainly consider government behavior and informal constraints in land improvement, including government investment, government subsidies, and social norms. Most scholars believe that financial incentives, such as cost compensation and fair subsidies, are the most effective tools to alleviate the constraints of farmers’ environmental behavior [43,47,48]. Therefore, if the government provides relevant subsidies for farmers to increase the fertility of arable land, it can encourage farmers to improve the land. If the government adopts related projects to improve the quality of arable land, it may squeeze out farmers’ private investment to a certain extent [7]. In the questionnaire, "For land improvement, what do people around you do" represents social norms. If surrounding farmers improve land, then informal constraints and demonstration effects will be formed, prompting farmers to improve the soil. 

## 3. Results

As the XPCC adjusted land for less than two years and about 10% of farmers have been in possession of land for less than a year, the real intentions of some farmers have not been reflected. So, we removed a sample of land that had been farmed for less than a year (including a year).

### 3.1. Basic Results

Table 5 reports the regression results for the probability of land improvement by using an ordered probit model, IV ordered probit, conditional mixed process (CMP), and control function (CF). Among them, Model I does not consider the endogeneity; Model II, Model III, and Model VI are the result of considering the endogeneity; Model V and Model VI add the technical experience, the role of production inertia, and its effect on the endogeneity are investigated.

In Model II, the instrumental variable was positively correlated with the endogenous variable at one percent level in the first-stage regression, and the F statistic is also significant at the one percent level (see as Appendix A Table A1), which indicates that there is no problem of a weak tool variable. In addition, children are less likely to continue farming if they have a higher degree of child discipline, a village cadre, a higher level of income, and a greater sense of social norms. This shows that the higher the level of a family’s human capital, social capital, and economic capital, the less likely the children are to engage in agricultural production. If farmers are risk-averse and have received technical guidance from the government, their children are more likely to continue farming. There are also regional differences in whether children continue to farm. In contrast, the children of the First Division were more likely to continue farming than the children of the Seventh Division.

The Atanhrho of Model III is significant at the one percent statistical level, which indicates that Equations (8) and (10) need to be estimated jointly, and the endogenous problem cannot be ignored. The residual from child farming (λ) in Model VI was significant at the one percent statistical level, which indicates the rejection of the assumption that children farming is an exogenous variable. The results from Model I to Model VI show that the validity of the key variables will be affected if the endogenous problem is ignored. After considering endogenesis, the influence of key variables was more significant, but the direction of influence did not change.

According to the results of Model V and Model VI, the goodness of fit of the model is improved when the technical experience variables are added. And this variable alleviates the endogeneity of the model, because the Atanhrho in the CMP model is not significant, and the coefficients of the two models are basically the same, which means that the joint estimation of Equation (8) and Equation (10) is rejected. 

The coefficient estimates for land tenure in Table 5 provide the primary basis for testing the research hypothesis. Farmers’ past experience of land readjustment has a significant positive impact on their decision to make land improvements. The possibility of improving the land of the farmers who have adjusted the land more than two times is significantly higher than that of the farmers who have not adjusted the land. As expected, there was a significant positive correlation between the remaining term of cultivation and the behavior of land improvement. After considering endogenesis, there was a significant negative correlation between child farming and land improvement behavior, but the negative correlation was not significant when the technical experience was included.

Besides land tenure, capital and technology are the most important factors that restrict farmers to improve their land. Consistent with the hypothesis, if farmers have financial constraints, then the possibility of improving the land will be greatly reduced. Meanwhile, if the farmer had the technical experience to improve the land in the past or has received the related technical service of the enterprise, that is, the farmer has less technical restrictions, then the possibility that the farmer will improve the land will be higher.

In addition, we found that farmers in the Seventh Division were significantly more likely to improve their land than those in the First Division. According to the data of the two, the average value of age, planting experience, planting area, specialization, and technical experience of the First Division farmers are significantly lower than those of the Seventh Division.

### 3.2. Heterogeneity Analysis

Table 6 presents the heterogeneous effects of land tenure on land improvement by categories of region and age, based on Model V in Table 5. In age-based regression, the mean age of 42 years was taken as the cut-off point, and the samples under 42 years of age and over 42 years of age were regressed, respectively. 

According to the grouping results, the remaining terms have a significant positive effect on the First Division farmers and the farmers over 42 years of age as shown in Table 5, and the marginal effect is higher than the corresponding group. This suggests that only by ensuring that farmers have a longer-term land tenure will they be more likely to improve their land in the First Division. As the land use term of the older farmers comes to an end, the impact of land tenure on farmers over 42 years of age is more significant. The variable of child farming is not significant in all models, which means that the intergenerational transfer of land does not affect the behavior of farmers in their own careers. The variable of the number of readjustments has a significant positive effect on the First Division farmers and on the farmers under 42 years of age. This also suggests that experience and motivation are more important for the First Division and for farmers under 42 years of age to improve their land.

Given the limited rationality of farmers, it is unlikely that they would undertake improvements if they did not have access to the relevant technologies or services, even if land tenure was stable. Furthermore, we investigated the moderating effect of technical constraints on the group with an insignificant effect of the remaining years variable (shown in columns 4 and 6). The results show that the interaction between the remaining years and technical constraints variables is significant, which indicates that the above-mentioned moderating effect does exist.

## 4. Discussion

In this paper, the number of readjustments, remaining term, and child farming were selected to represent the past, present and future stability of land tenure. The results are somewhat inconsistent with existing studies.

First of all, whether the land tenure was stable in the past does not affect the current behavior of farmers, because the number of land readjustment in the past is in a significantly positive correlation with the behavior of farmers to improve land. This is somewhat different from previous similar studies, which have suggested that past experiences of land instability will lead farmers to reduce associated conservation practices [24,29]. Generally, if farmers frequently experience land readjustment, it may lead to their cognitive instability of land tenure. Then, we find that the farmers whose land is frequently adjusted are usually older, more experienced, have larger planting areas, and are more specialized in planting cotton. The influence of experience and motive factors in land improvement outweighs that of tenure. Our result indicates that recent land tenure changes in the XPCC have reduced the tenure insecurity so that farmers have a relatively stable expectation of current land tenure, and past land changes are not so important. This also means that farmers have a high degree of trust in the government and its policies.

Secondly, the remaining term is positively correlated with the farmers’ behavior of land improvement, which indicates that the longer-term tenure (maybe five years or more) is more important to the farmers. More studies suggest that more than five years means relatively long-term property rights. Our results demonstrate that farmers with a tenure of five years or more are more likely to improve their land. As the government has undertaken relatively long-term cultivated land conservation investment (such as low- and medium-yield farmland transformation, high-standard farmland construction, and other engineering measures), and the payback period of investment carried out by farmers is not that long. The specific results are consistent with some similar studies that take the land tenure term or its expectation as a proxy variable, such as whether to continue farming in the next five years and the remaining contract period [24,25,49,50,51]. However, such variables are not always so significant in similar studies and vary with different conservation practices [4,7,24].

Finally, farmers’ behavior of land improvement is not related or even negatively related to their children’s continued farming, which indicates that the intergenerational transfer of land tenure is not the key factor affecting farmers’ behavior. This is not in line with expectations. If the land tenure expires and the farmer’s children continue farming, that means a longer planning period, and more stable land tenure should encourage more investment in land improvement. In countries where land has not been privatized, legacy rights are often used as a proxy variable in African studies, and the results are quite different [5,22,26]. However, few studies consider the issue of children inheriting farmland in China. Based on the sample data, it can be found that farmers with higher educational backgrounds and grassroots cadres are less likely to let their children continue farming. If children continue to farm after the contract expires in the XPCC, it not only indicates the stability of land tenure but also that there are no grassroots cadres in farmer households, and their children’s education level is relatively low. The low level of household human capital and social capital may hinder the use of relevant land improvement technologies, which may exceed the positive impact of tenure. Therefore, under the background that many Chinese scholars are discussing whether to carry out a “permanent tenancy system” and endow the right of land inheritance, endowing the right of land contract and management with the right of inheritance does not seem to promote the conservation of farmland.

Although a stable land contracting policy encourages farmers to protect arable land, its role may be relatively limited. We can see that as many as 45.39% of farmers have taken measures to improve their land, even though the land tenure is not as stable as after land readjustment. This suggests that factors other than property rights, such as government technology diffusion, and a free supply of farm manure may have played an important role in past land improvements. The corps adopted an administrative top-down technology extension system, with each grassroots farm having administrative personnel dedicated to solving technical problems in agricultural production. In addition, some farms will provide farm manure and other materials free of charge to their farmers, but these materials require farmers to spend their own manpower, material resources, and time to obtain. The above-mentioned material does not mean that all farmers will improve the land; it depends on the farmers’ own willingness.. Because of the limitation of the farmers’ own ability, their actual behavior choice is limited. Government intervention provides sufficient technical information for farmers to improve land, which greatly alleviates farmers’ cognitive constraints, and makes farmers develop sustainable improvement behavior. With the reduction of the government’s administrative intervention after the land reform, it is necessary to consider a market-oriented way to encourage farmers to improve the land.

## 5. Conclusions

In this article, we developed a framework to examine the relationship between land tenure and land improvement behavior in state-owned farms in Xinjiang (XPCC) of China. The land tenure considered includes the number of land adjustments experienced, remaining term for cultivation, and whether the children will farm after the contract expires, which represents the stability of land tenure in the past, the present, and the future. The empirical results are somewhat different from our hypothesis. Specifically, the more times the land is adjusted, the more likely the farmer is to improve the land. As a rich experience, a larger scale and a high degree of specialization are associated with the greater number of land adjustments experienced by farmers, and these factors have a greater impact than property rights, which lead to inconsistent conclusions. If the child continues farming after the contract expires, the farmer is less likely to improve the land, as the child’s continued farming is usually associated with the family’s lower level of human and social capital. Consistent with the hypothesis, if the farmer can continue to cultivate the land after five years, then he is more likely to improve the land.

Our results show that the legal and actual stability of land tenure has been strengthened, which plays a certain role in encouraging farmers to conserve cultivated land. However, institutional and technical factors play a key role in the protection of cultivated land. The government’s administrative technology promotion alleviates farmers’ limited rationality, enables them to have enough relevant knowledge to take measures to conserve cultivated land and benefit from it, and finally forms the sustainable cultivated land protection behavior.

Therefore, in order to make farmers improve the land, we should first consider the establishment of a market-oriented technology extension system. With the reduction of government administrative intervention, it is necessary for the government to guide the establishment of a top-down technical service chain for agricultural enterprises, agricultural distributors, and farmers by relying on the innovation of the product service mode of the agricultural enterprises. Secondly, the government needs to improve the land contract withdrawal mechanism. In view of the increase or decrease of cultivated land capacity, the contract should make clear the standard of compensation (punishment) and how to compensate (punish) so as to make the management subject aware of the realizable value of cultivated land conservation. Thirdly, the government needs to adopt policies to guide the optimization of green credit services to ease the financial constraints of farmers. Of the sample, 65.64 % had access to credit, and 71.38% were still in financial difficulties. At present, the XPCC launched a one-year credit service for farmers, which can greatly ease the financial constraints of farmers, but the XPCC still needs to expand the coverage and diversity of credit services.

This study also suffers from limitations. First, we only discussed the adoption of farmers; the degree of adoption, such as cost and number of years of adoption, was not considered. Because the farmer’s land improvement behavior includes many different improvement measures, the adoption interval and adoption cost of these measures are quite different. The summation of it is quite complex, and we neglected this data collection. Secondly, although we have specific land improvement measures and the impact of land tenure on the adoption of these different measures varies, we did not discuss them further due to the lack of information on prices, accessibility, and so on.

## Figures and Tables

**Table 1:** Description of land adjustment and land tenure.

	Frequency	Proportion		Frequency	Proportion
The number of land adjustments experienced			The latest adjustment to land changes		
0 time	396	38.41%	Area change	705	69.19%
1 time	302	29.29%	Plot change	131	12.86%
2 times	129	12.51%	No change	286	28.07%
More than 3 times	204	19.79%	Number of plots	1019	100%
Number of plots	1031	100%	Years left		
The way of dealing with when contract expires			Less than 3 years	124	12.03%
Children continue to farm	356	34.53%	3 to 5 years	97	9.41%
Relatives continue to farm	32	3.10%	5 to 10 years	207	20.08%
Return to regimental farm	622	60.33%	10 to 20 years	360	34.92%
Others	21	2.04%	More than 20 years	243	23.57%
Number of plots	1031	100%	Number of plots	1031	100%

Note: “The latest adjustment to land changes” is multiple choice, and there are missing values, so its frequency does not add up to 1031.

**Table 2:** A cross-analysis on the consistency of farmers’ behavior in land improvement.

Improved Land after 2017	Improved Land before 2017		
	Improved	Unimproved	Total
improved	451 (96.37%)	65 (11.55%)	516 (50.05%)
under consideration	16 (3.42%)	463 (82.24%)	479 (46.46%)
unimproved	1 (0.21%)	35 (6.22%)	36 (3.49%)
number of plots	468 (100%)	563 (100%)	1031 (100%)

Note: Before the reform in 2017, the contract period could not exceed 30 years, and the contract was signed with employees in installments (usually 5 years). In 2017, the XPCC carried out land readjustment and land titling, with land distributed evenly and contracts limited to the retirement age. Land tenure is more stable than it was before the land reform. Before the land reform, the productive inputs (including land improvement) other than the “Five unification” were generally chosen by farmers according to the actual situation, but the farm will carry out technology extension. After the land reform, it was entirely up to the farmers to make their own choices. The numbers outside the parentheses are frequency numbers, and the numbers in parentheses are percentages.

**Table 3:** Description of farmers’ land improvement measures.

Land Improvement	All Plots		First Division Plots		Seventh Division Plots	
	Frequency	Proportion	Frequency	Proportion	Frequency	Proportion
Organic fertilizers	324	60.79%	160	67.80%	164	55.22%
Microbial fertilizers	184	34.52%	63	26.69%	121	40.74%
Formula fertilization	192	36.02%	87	36.86%	105	35.35%
Compound fertilizers with higher organic matter	200	37.52%	60	25.42%	140	47.14%
Soil amendments	104	19.51%	49	20.76%	55	18.52%
Other	80	15.01%	49	20.76%	31	10.44%
Number of plots	533		236		297	

Note: “Land improvement measures” is multiple choice.

**Table 4:** Variable Selection, Definition, and Prediction.

Variable	Definition	Mean	SD	Prediction
Dependent variable				
Land improvement behaviors	Whether the land has improved after the latest land adjustment in 2018 (2 = yes, 1 = under consideration, 0 = no)	1.48	0.57	
Land tenure security				
Number of readjustments	Number of land adjustments experienced by farmers (3 = 2 times or more, 2 = 1 time, 1 = 0 time)	2.00	0.84	-
Remaining term	Whether the remaining farming years are more than 5 years (1 = yes, 0 = no)	0.85	0.35	+
Child farming	When the contract expires, the children continue to farm (1 = yes, 0 = no)	0.34	0.47	+
Basic family characteristics				
Age	Age of the household head (years)	42.47	9.04	+/-
Education	The highest degree for all family members (1 = junior high school and below, 2 = senior high school, 3 = junior college, 4 = university graduate or above)	2.47	1.10	+
Grassroots cadres	Families have grassroots cadres (1 = yes, 0 = no)	0.17	0.38	+
Risk attitude	Choose alternative production technology (3 = high risk and high return, 2 = medium risk and medium return, 1= low risk and low return)	1.97	0.71	+
Cotton planting characteristics				
Experience	Number of years that farmers have grown cotton (years)	12.64	17.85	+/-
Area planted	Area of cotton field (mu)	58.33	31.66	+
Specialization	The percentage of cotton revenue in total revenue (%)	0.62	0.33	+
Economic factors				
Profitability	Farmers’ perception of the benefits of land improvement (4 = great benefits, 3 = a little benefit, 2 = uncertain, 1 = no benefit)	3.74	0.55	+
Income	Average family income (1 = 40,000 yuan and below, 2 = 40,000–100,000-yuan, 3 = over 100,000 yuan)	1.52	0.57	+
Financial constraints	Families have financial constraints on land improvement (1 = yes, 0 = no)	0.71	0.45	-
Technical factors				
Government assistance	Obtained government training or technical personnel guidance (1 = yes, 0 = no)	0.64	0.48	+
Enterprises services	Obtained technical services from the company or sales staff (1 = yes, 0 = no)	0.44	0.50	+
Technical constraints	There are technical or service constraints on land improvement (1 = yes, 0 = no)	0.55	0.50	-
Technical experience	Land improvement techniques have been used before land adjustment in 2018 (1 = yes, 0 = no)	0.47	0.50	+
Institution factors				
Government investment	The government invested in engineering measures to improve farmland quality (1 = yes, 0 = no)	0.10	0.30	-
Government subsidies	The government provided subsidies for improving farmland quality (1 = yes, 0 = no)	0.31	0.46	+
Social norms	What are the people around you doing about land improvement? (4 = all do that, 3 = most people do that, 2 = a few people do that, 1 = no one does that)	3.15	0.78	+

Note: “+” means positive impact, “-” means negative impact, “+/-” means uncertain impact.

**Table 5:** Determinants of land improvement behaviors.

Variable	Model I Oprobit		Model II IV-oprobit		Model III CMP		Model VI CF (2SCML)		Model V Oprobit		Model IV CMP	
	Coeff	SD	Coeff	SD	Coeff	SD	Coeff	SD	Coeff	SD	Coeff	SD
Land tenure												
Number of readjustments												
1 time	−0.002	0.109	−0.018	0.109	−0.015	0.096	−0.019	0.109	0.245 *	0.145	0.245 *	0.146
2 times or more	0.225 **	0.109	0.228 **	0.110	0.190 *	0.104	0.224 **	0.110	0.291 **	0.145	0.291 **	0.146
Remaining term	0.236 *	0.125	0.321 **	0.128	0.273 **	0.120	0.316 **	0.128	0.326 **	0.163	0.326 **	0.165
Child farming	−0.146	0.094	−1.511 ***	0.365	−1.257 ***	0.154	−1.452 ***	0.368	−0.142	0.123	−0.142	0.489
Basic family characteristics												
Age	−0.002	0.005	−0.004	0.005	−0.003	0.005	−0.004	0.005	−0.007	0.007	−0.007	0.007
Education												
senior high school	0.012	0.125	−0.046	0.127	−0.039	0.108	−0.041	0.127	0.033	0.167	0.033	0.168
junior college	0.035	0.126	−0.164	0.136	−0.117	0.115	−0.142	0.135	0.094	0.167	0.094	0.179
university graduate or above	−0.086	0.141	−0.345 **	0.157	−0.287 **	0.134	−0.336 **	0.157	−0.202	0.187	−0.202	0.207
Grassroots cadres	−0.110	0.131	−0.258 *	0.137	−0.215 *	0.125	−0.252 *	0.137	0.044	0.173	0.044	0.181
Risk attitude												
Risk neutral	0.235 **	0.106	0.289 ***	0.107	0.252 ***	0.094	0.287 ***	0.107	0.177	0.140	0.177	0.141
Risk preference	0.244 **	0.122	0.348 ***	0.125	0.297 **	0.116	0.341 ***	0.125	0.110	0.161	0.110	0.165
Cotton planting characteristics												
Experience	0.002	0.002	0.001	0.002	0.001	0.002	0.001	0.002	0.000	0.003	0.000	0.003
Area planted	0.003	0.002	0.004 **	0.002	0.003 **	0.002	0.004 **	0.002	−0.002	0.002	−0.002	0.002
Specialization	−0.134	0.138	−0.141	0.139	−0.121	0.131	−0.139	0.139	−0.135	0.182	−0.135	0.182
Economic factors												
Profitability												
uncertain	−1.342	0.946	−1.415	0.959	−1.194	0.784	−1.415	0.953	−0.514	1.311	−0.514	1.322
a little benefit	−1.061	0.936	−1.120	0.949	−0.941	0.777	−1.119	0.943	−0.471	1.306	−0.471	1.317
great benefits	−1.098	0.933	−1.127	0.945	−0.945	0.779	−1.126	0.939	−0.574	1.303	−0.574	1.314
Income												
40,000–100,000-yuan	−0.030	0.094	−0.047	0.095	−0.026	0.088	−0.045	0.095	0.034	0.125	0.034	0.125
over 100,000 yuan	−0.154	0.235	−0.216	0.236	−0.229	0.211	−0.216	0.236	0.020	0.294	0.020	0.295
Financial constraints	−0.392 ***	0.104	−0.448 ***	0.105	−0.379 ***	0.099	−0.447 ***	0.105	−0.419 ***	0.142	−0.419 ***	0.143
Technical factors												
Government assistance	0.057	0.092	0.162 *	0.097	0.132	0.089	0.158	0.097	0.062	0.121	0.062	0.125
Enterprises services	0.187 **	0.089	0.174 *	0.090	0.146 *	0.085	0.173 *	0.090	0.271 **	0.119	0.271 **	0.120
Technical constraints	−0.392 ***	0.092	−0.422 ***	0.093	−0.355 ***	0.089	−0.417 ***	0.093	−0.336 ***	0.123	−0.336 ***	0.123
Technical experience									2.945 ***	0.152	2.945 ***	0.157
Institution factors												
Government investment	0.091	0.149	0.028	0.150	0.036	0.141	0.036	0.150	0.129	0.194	0.129	0.196
Government subsidies	0.241 **	0.099	0.324 ***	0.102	0.272 ***	0.094	0.319 ***	0.102	−0.061	0.134	−0.061	0.138
Social norms												
a few people do that	0.215	0.277	0.226	0.278	0.192	0.233	0.215	0.278	−0.120	0.329	−0.120	0.330
most people do that	0.937 ***	0.271	0.851 ***	0.273	0.708 ***	0.238	0.844 ***	0.273	0.146	0.326	0.146	0.327
all do that	1.020 ***	0.276	0.910 ***	0.278	0.779 ***	0.250	0.906 ***	0.278	0.296	0.333	0.296	0.335
Residual from child farming							1.401 ***	0.381				
atanhrho_12					0.911 ***	0.179					0.038	0.302
Region variable	Control		Control		Control		Control		Control		Control	
*N*	873 ^1^		873		873		873		873		873	
Log likelihood	−626.024		−618.608		−1106.934		−619.234		−319.486		808.777	
LR chi2 or F-test	173.67 ***		188.497 ***		487.90 ***		187.25 ***		786.74 ***		544.46 ***	
Pseudo R^2^ or Adj R-squared	0.122		0.132				0.131		0.552			

Note: ***, **, * represent statistically significance at 1%, 5% and 10%, respectively. ^1^ Samples that had been cultivated for less than one year were excluded.

**Table 6:** Heterogeneity analysis.

Variable	Group by Region			GROUP BY AGE		
	First Division	Seven Division	Seven Division	<42	<42	≥42
Number of readjustments						
1 time	0.050 ** (0.024)	0.025 (0.035)	0.022 (0.034)	0.048 (0.030)	0.051 * (0.029)	0.023 (0.027)
2 times or more	0.047 * (0.027)	0.040 (0.031)	0.038 (0.032)	0.087 ** (0.038)	0.086 ** (0.038)	0.014 (0.024)
Remaining term	0.054 * (0.028)	0.050 (0.037)	0.053 (0.037)	0.031 (0.051)	0.040 (0.051)	0.053 ** (0.025)
Child farming	−0.016 (0.021)	−0.013 (0.030)	0.011 (0.031)	0.001 (0.027)	0.003 (0.027)	−0.034 (0.021)
Technical constraints	−0.029 (0.021)	−0.086 *** (0.028)	−0.091 *** (0.028)	−0.055 ** (0.027)	−0.035 (0.030)	−0.039 * (0.022)
Number of readjustments × Technical constraints			−0.006 (0.030)		0.002 (0.033)	
Remaining term × Technical constraints			−0.127 * (0.071)		−0.253 ** (0.109)	
Child farming × Technical constraints			−0.010 (0.061)		0.037 (0.052)	
Control variable	yes	yes	yes	yes	yes	yes
*N*	459	414	414	384	384	489
Log likelihood	−173.255	−129.024	−127.430	−148.604	−145.509	−155.043
LR or F	421.88 ***	352.17 ***	355.35 ***	340.69 ***	346.88 ***	476.21 ***
Pseudo R^2^ or Adj R^2^	0.549	0.578	0.582	0.534	0.544	0.606

Note: ***, **, * represent statistically significance at 1%, 5% and 10%, respectively. The standard deviation is in parentheses.

## Data Availability

Please refer to suggested Data Availability Statements in section “MDPI Research Data Policies” at https://www.mdpi.com/ethics.

## Appendix A

**Table A1:** Determinants of children farming.

	Coef.	St.Err.	*t*-Value	*p*-Value	[95% Conf	Interval]	Sig
Average child farming	1.751	0.218	8.020	0.000	1.323	2.178	***
Remaining years	0.213	0.133	1.610	0.108	−0.047	0.473	
Number of readjustments	0.004	0.056	0.080	0.938	−0.106	0.115	
Age	−0.005	0.005	−0.950	0.344	−0.016	0.006	
Education	−0.169	0.046	−3.710	0.000	−0.259	−0.080	***
Grassroots cadres	−0.412	0.136	−3.030	0.002	−0.678	−0.146	***
Risk attitude	0.141	0.062	2.260	0.024	0.019	0.264	**
Experience	0.000	0.003	−0.050	0.958	−0.006	0.006	
Area planted	0.002	0.002	1.100	0.270	−0.001	0.005	
Specialization	−0.161	0.135	−1.190	0.236	−0.426	0.105	
Region variable	−0.235	0.107	−2.200	0.028	−0.445	−0.025	**
Profitability	−0.09	0.085	−1.070	0.285	−0.256	0.075	
Income	−0.145	0.084	−1.730	0.084	−0.310	0.020	*
Financial constraints	−0.131	0.102	−1.280	0.200	−0.332	0.070	
Government assistance	0.221	0.097	2.280	0.022	0.031	0.411	**
Enterprises services	−0.047	0.092	−0.510	0.607	−0.227	0.132	
Technical constraints	0.001	0.093	0.010	0.989	−0.180	0.183	
Government investment	0.031	0.152	0.200	0.840	−0.267	0.328	
Government subsidies	0.153	0.099	1.540	0.125	−0.042	0.347	
Social norms	−0.103	0.062	−1.660	0.098	−0.226	0.019	*
Constant	0.091	0.457	0.200	0.843	−0.805	0.986	
*N*	988						
Log likelihood	−551.787						
LR chi2	165.852 ***						
Pseudo R^2^	0.131						
F value of the instrumental variable	9.17***						

Note: ***, **, * represent statistically significance at 1%, 5% and 10%, respectively.
